# Co-transcriptional splicing is delayed in the highly expressed thyroglobulin gene

**DOI:** 10.1242/jcs.263872

**Published:** 2025-03-19

**Authors:** Simon Ullrich, Iliya Nadelson, Stefan Krebs, Helmut Blum, Heinrich Leonhardt, Irina Solovei

**Affiliations:** ^1^ Faculty of Biology, Ludwig Maximilians University Munich, 82152 Planegg-Martinsried, Germany; ^2^ Laboratory for Functional Genome Analysis (LAFUGA), Gene Center, Ludwig Maximilians University Munich, 81377 Munich, Germany

**Keywords:** Transcription, Splicing, Single-cell analysis, *Tg* gene, Intron splicing delay

## Abstract

Transcription of the majority of eukaryotic genes is accompanied by splicing. The timing of splicing varies significantly between introns, transcripts, genes and species. Although quick co-transcriptional intron removal has been demonstrated for many mammalian genes, most splicing events do not occur immediately after intron synthesis. In this study, we utilized the highly expressed *Tg* gene, which forms exceptionally long transcription loops, providing a convenient model for studying splicing dynamics using advanced light microscopy. Using single-cell oligopainting, we observed a splicing delay occurring several tens of kilobases downstream of a transcribed intron, a finding supported by standard cell population analyses. We speculate that this phenomenon is due to the abnormally high transcriptional rate of the *Tg* gene, which might lead to a localized deficiency in splicing factors and, consequently, delayed spliceosome assembly on thousands of nascent transcripts decorating the gene. Additionally, we found that, in contrast to what is seen for short introns (<10 kb), the long *Tg* intron (>50 kb) is spliced promptly, providing further support for the idea that intron length might modulate splicing speed.

## Teaser

**Highlighted Article:** Using the greatly upregulated thyroglobulin gene as a model, this study demonstrates that, in highly expressed genes, intron splicing might be delayed for several tens of kilobases.

## INTRODUCTION

Eukaryotic genes differ from prokaryotic genes because they have intragenic non-coding sequences, or introns, which play a crucial role in regulation of gene expression, gene evolution and protein heterogeneity. This eukaryotic innovation, however, comes at the cost of a complex, energy-consuming and tightly regulated process of intron splicing (Alpert et al., 2017; de Almeida and Carmo-Fonseca, 2008; Muller-McNicoll and Neugebauer, 2013). Despite substantial progress in splicing research, the timing of splicing events remains a subject of ongoing investigation and debate (Eichenberger et al., 2023; Gordon et al., 2021; Han et al., 2011; Merens et al., 2024).

The spliceosome complex identifies key genomic elements that define an intron, such as the splice donor, splice acceptor and branchpoint, to initiate the splicing process. After an intron is transcribed, the spliceosome excises it, typically with RNA polymerase II positioned tens of nucleotides downstream of the 3′ border of the intron (Oesterreich et al., 2016). Although co-transcriptional intron removal has been demonstrated for many mammalian genes (Brugiolo et al., 2013), most splicing events do not occur immediately after the synthesis of an intron (Merens et al., 2024). Over the past decade, several deviations from the canonical co-transcriptional splicing model have been described.

One example is post-transcriptional splicing, where introns are removed in any order after pre-mRNA polyadenylation (Brody et al., 2011; Coté et al., 2024; Drexler et al., 2020; Gaidatzis et al., 2015; Zeng et al., 2022). Another widespread variation is intron retention, in which one or more specific introns are preserved in polyadenylated RNA to regulate the release of mRNA into the cytoplasm (Braunschweig et al., 2014; Jacob and Smith, 2017; Pimentel et al., 2016). Finally, canonical splicing can be supplemented with recursive splicing (Zhang et al., 2018), a process of intron removal via multiple rather than a single splice step, with apparently stochastic splice site selection (Duff et al., 2015; Sibley et al., 2015; Wan et al., 2021).

**Fig. 1.**
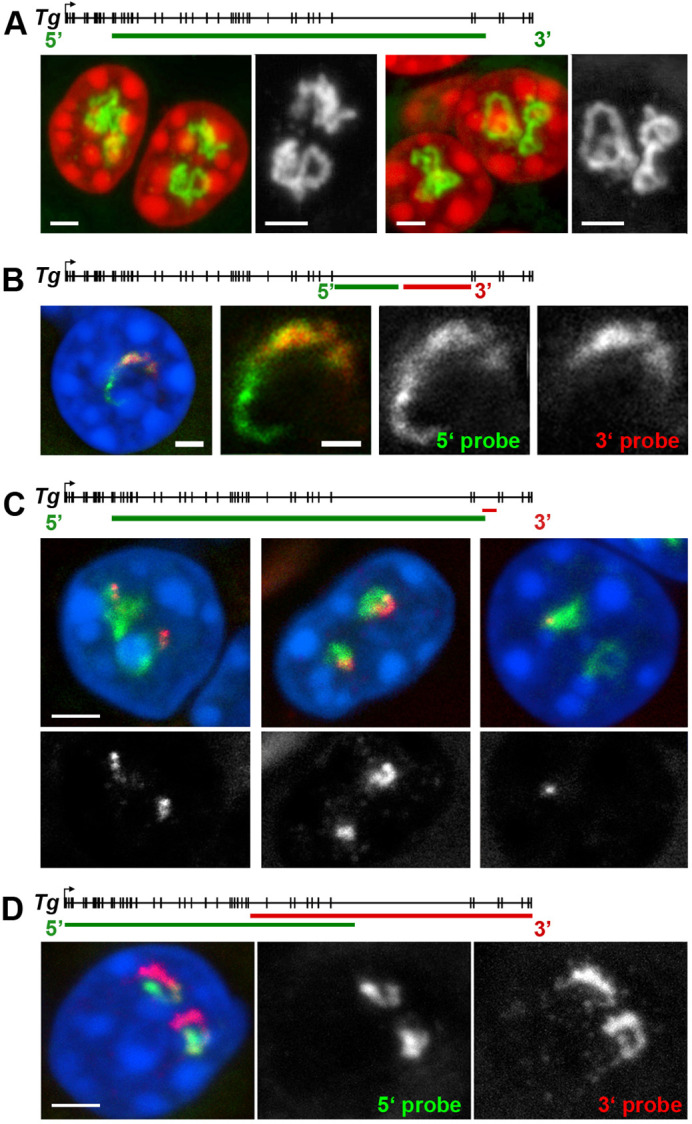
**Great extension of the *Tg* TLs allows transcription analysis using light microscopy.** (A) Several examples of *Tg* TLs (green) within thyrocyte nuclei (red) detected by RNA-FISH using a genomic BAC probe encompassing ∼150 kb of the 180 kb long *Tg*. Note the volatile and strongly convoluted shape of TLs. (B) Labeling of intron 40 by RNA-FISH with oligoprobes for 5′ (green) and 3′ (red) halves of the intron. 5′ probe labels the entire intron, whereas the 3′ probe labels only half of the intron. The increasing thickness of the nRNAs matrix, thin at the 5′ end and thick at the 3′ end, reflects the transcription direction. (C) The oligoprobe for the intron 44 at the 3′ end of the gene (red) in combination with BAC probe for the whole loop (green) after RNA-FISH. Three examples demonstrate transcriptional bursting: intron signals can have similar sizes (left), different sizes (mid), or signal can be on only one of the alleles (right). (D) Labeling of the Tg TLs with two BAC clones encompassing 5′ (green) and 3′ (red) halves of the loops by RNA-FISH. Although the probes overlap in the middle of the gene, on a global scale, they label the TLs sequentially as a result of co-transcriptional splicing. Every panel is accompanied by schematics above the images illustrating the coverage of the gene with probes. Images are projections of short confocal stacks; nuclei are counterstained with DAPI. RGB panels are supplemented with grey scale images of the RNA signals. Images are representative of hundreds of experimental repeats. Scale bars: 2 µm (A, main images; C,D); 1 µm (B) and 0.5 µm (A,B, magnified views).

We recently demonstrated that highly expressed long genes expand from their harboring loci and form microscopically resolvable transcription loops (TLs) (Leidescher et al., 2022). This TL extension has been attributed to the dense decoration of the gene with thousands of polymerases with attached nascent ribonucleoprotein granules (nRNPs), which impart intrinsic stiffness to the gene axis. In essence, the extent of TL expansion is influenced by the size of the attached nRNPs, which become increasingly voluminous over long exons or long introns, linking TL expansion to splicing dynamics. One of the studied genes, the thyroglobulin (*Tg*) gene, is particularly highly upregulated, far surpassing the expression levels of other genes, including many housekeeping and tissue-specific genes (Leidescher et al., 2022; Ullrich et al., 2023). Notably, although the *Tg* gene is not very long (180 kb in mice), it forms exceptionally long TLs that extend several microns into the nucleoplasm (Fig. 1A).

The substantial extension of the *Tg* gene is somewhat counterintuitive, given its conventional organization – it contains 48 exons ranging from 66 to 233 nt and introns ranging from 197 to 9375 nt, with only one large intron of ∼54 kb in length. The *Tg* mRNA is only 8.4 kb long, and assuming quick co-transcriptional splicing, the lengths of nascent RNAs associated with *Tg* TLs should progressively decrease after each exon. This would result in smaller nRNPs decorating the gene, which should, in theory, permit the gene axis coiling and thus limit gene expansion. However, the *Tg* TLs expand substantially more compared to genes with long exons (e.g. *Ttn*) or long introns (e.g. *Cald1*) (Leidescher et al., 2022). One plausible explanation for the remarkable extension of *Tg* TLs is a delay in intron splicing. Such a delay would result in long nRNAs containing not only exons but also multiple unspliced introns, leading to the formation of bulky nRNPs.

The *Tg* gene is exclusively expressed in thyrocytes, the secretory epithelial cells organized into closed follicles within the thyroid gland. When isolated from tissue and incubated *in vitro*, thyrocytes rapidly lose their identity and silence the *Tg* gene (Ullrich et al., 2023). Consequently, modern methods for studying splicing dynamics, such as live-cell imaging (Martin et al., 2013) or metabolic labeling of nRNAs (Schwalb et al., 2016), and POINT technology (Sousa-Luis et al., 2021) etc., cannot be applied in this context.

To test our hypothesis regarding delayed splicing of *Tg* nascent RNAs (nRNAs), we employed several methods compatible with thyroid tissue analysis. Standard cell population techniques, such as PCR and nanopore sequencing, did not provide sufficient information. Therefore, we conducted single-cell analyses using oligopainting to label consecutive *Tg* introns, followed by rigorous formal analysis of three-dimensional (3D) images. Collectively, the data indicate a significant delay in the splicing of *Tg* introns, which we hypothetically attribute to the exhaustion of local splicing machinery caused by the high transcriptional activity of the gene.

## RESULTS

The remarkable length of *Tg* TLs (Fig. 1A) makes the gene a unique and attractive model for studying transcription and splicing through light microscopy, as demonstrated by the following three examples. First, RNA-fluorescence *in situ* hybridization (FISH) with differential labeling of the two halves of the 54 kb *Tg* intron enables us to observe the dynamics of intron splicing. Oligoprobes targeting the 3′ half label the second portion of the intron as anticipated, whereas oligoprobes for the 5′ half label the entire intron (Fig. 1B), suggesting that this long intron is likely excised as an entire piece. Admittedly, given that the resolution of light microscopy does not allow for the analysis of individual nascent RNAs, we cannot completely rule out the possibility that some nascent transcripts undergo recursive splicing. However, the strong signal over the intron and gradient of nRNA suggest that, if recursive splicing does occur, it is not widespread. Second, RNA-FISH using oligoprobes targeting nRNA over 5 kb region of the last *Tg* intron reveals transcriptional bursting of the gene. Nuclei displayed oligoprobe signals that were distributed equally among three categories: similar in size on both alleles, different in size between alleles, or present on only one allele (Fig. 1C). The absence of RNA signals suggests that this intron, at the specific time of observation, remained in a transcriptional pause, whereas signals of differing sizes reflect nonsynchronous transcriptional bursting. Third, our previous studies of TLs formed by long highly expressed genes have demonstrated co-transcriptional splicing but only on a large scale. RNA-FISH with two bacterial artificial chromosome (BAC) probes, primarily targeting 5′ and 3′ introns, sequentially labels the TLs – 5′ introns are extensively spliced out as transcription proceeds toward the 3′ end of the gene, rendering them practically undetectable with the 5′ probe (Fig. 1D; see also Leidescher et al., 2022; Ullrich et al., 2023). Undeniably, owing to the limited resolution of FISH with BAC probes, this experiment does not allow for a detailed assessment of splicing dynamics.

### RNA signals of *Tg* introns are disproportionally large

**Fig. 2.**
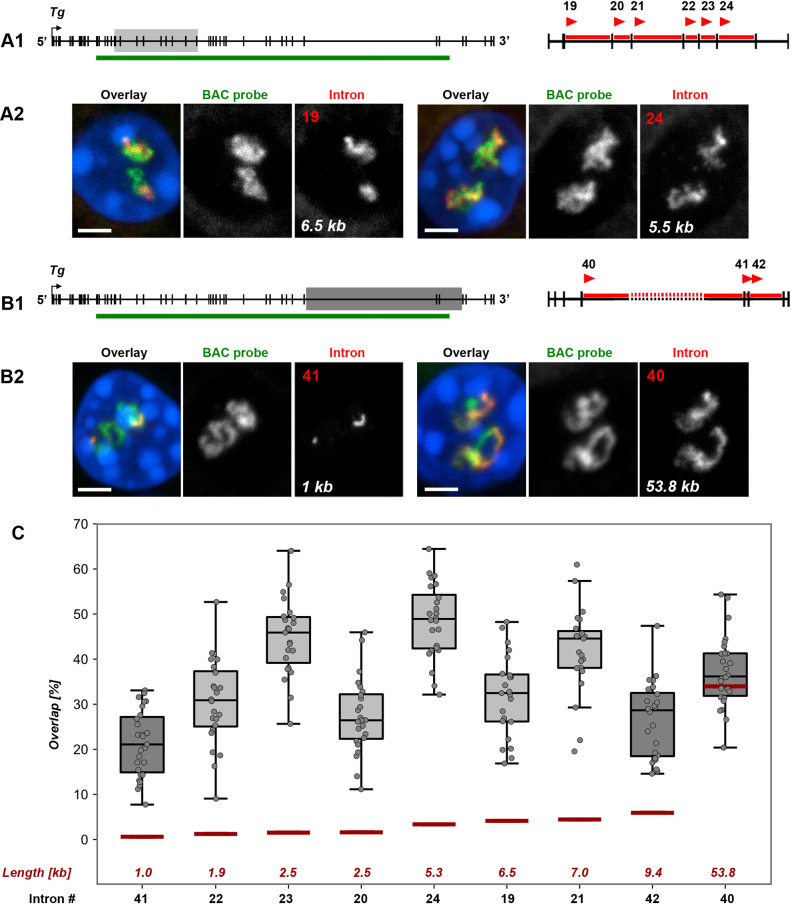
**RNA signals from single *Tg* introns are disproportionally large in comparison to RNA signal of a genomic probe.** (A,B) Pairwise comparison of RNA signals of single intron versus BAC probe (153 kb). In the gene schematics, sampled regions are marked with light-grey rectangle in the 5′ end (A) and with the dark-grey rectangle in the 3′ end (B). The green lines indicate coverage by the BAC probe; on the magnified views of the sampled regions, the arrowheads mark the 5′ end of introns, for which oligoprobes were designed; numbers of introns are indicated above arrowheads. Representative images of thyrocytes after RNA-FISH with pairwise probes (BAC, green; introns, red) are shown below the schematics; lengths of introns are indicated on the grey scale intron panels. For clarity, RGB images are supplemented with grey scale images of RNA signals. Scale bars: 2 µm. For more examples of nuclei in this experiment, see Fig. S1. (C) Boxplots showing the overlap ratio of intron signals with the BAC probe from both gene regions. Introns are arranged on the *x*-axis according to their size, from smallest to largest; intron numbers indicated below and their lengths – above the *x*-axis. Thick lines within boxes indicate the medians; boxes show 25th and 75th percentiles; whiskers represent the smallest and the largest values within 1.5 times the interquartile range from the quartile 1 and quartile 3 boundaries; gray dots are individual data points. Analysis of 25 nuclei from thyroids of three different mice. Note that the signal overlap of similarly sized introns can be significantly different, as it is the case for intron 20 and intron 23, both 2.5 kb in size (*P*<0.001). On the contrary, introns that differ in their size, such as intron 23 (2.5 kb) and intron 24 (5.5 kb), can show similar overlap ratios (*P*>0.05, one-way ANOVA with Tukey's multiple comparisons post hoc). Expected overlap proportions based on the genomic size of the probes are marked with red lines. Note that only in case of the long intron 40, expected and measured overlaps are similar; for all the other introns, the measured overlap exceeded the expected value by 6- to 10-fold.

To study splicing dynamics at higher resolution, we labeled individual introns with oligoprobes using signal amplification by exchange reaction (SABER)-FISH (Kishi et al., 2019). The oligoprobes were designed to target the first 1–3 kb of each intron to enable labeling of the entire intron. We selected six sequential introns ranging in size from 2 to 7 kb, located at the 5′ end of the gene (Fig. 2A), and three introns at the 3′ end of the *Tg* gene, including the longest intron 40, which has a length of 54 kb (Fig. 2B). Each intron was hybridized pairwise with a reference BAC probe in RNA-FISH. The BAC probe labeled nRNAs, including introns and exons, along a 153 kb region in the middle of the gene. Surprisingly, visual pairwise comparisons of the single intron signals with the BAC signal revealed disproportionately large signals for all introns except intron 40 (Fig. 2A,B; Fig. S1A,B).

To quantify signal disproportionality, we developed a dedicated workflow for 3D image analysis of confocal stacks. The program automatically segments and separates nuclei, identifies and segments RNA signals, corrects for chromatic shift between channels, and calculates the overlapping volume (*V*) between BAC and intron signals as the ratio *V*_overlap_/(*V*_intron_+*V*_BAC_) (see the Materials and Methods for details). Because RNA signals from the two alleles within a single nucleus are often inseparable and can vary in size owing to transcriptional bursting, the overlapping volume was calculated for both alleles together. This quantitative analysis confirmed the striking disproportionality of intron signals (Fig. 2C).

Based on the genomic sizes of the probed regions, the expected overlap proportion should correspond to the calculated intron-to-BAC genomic length ratio, as indicated by the dark red lines at the bottom of the graph in Fig. 2C. However, the observed overlap was much higher ranging from 26% to 49%. For example, the expected overlap between intron 21 (7 kb) and the BAC (spanning 153 kb of the gene), is ∼4%, but the measured overlap was over 40% (Fig. 2C). Interestingly, and somewhat counterintuitively, we observed a significantly larger overlap for short introns (1–10 kb), but not for the longest intron 40, which had an overlap value close to the expected ratio (i.e. ∼35%; Fig. 2C).

**Fig. 3.**
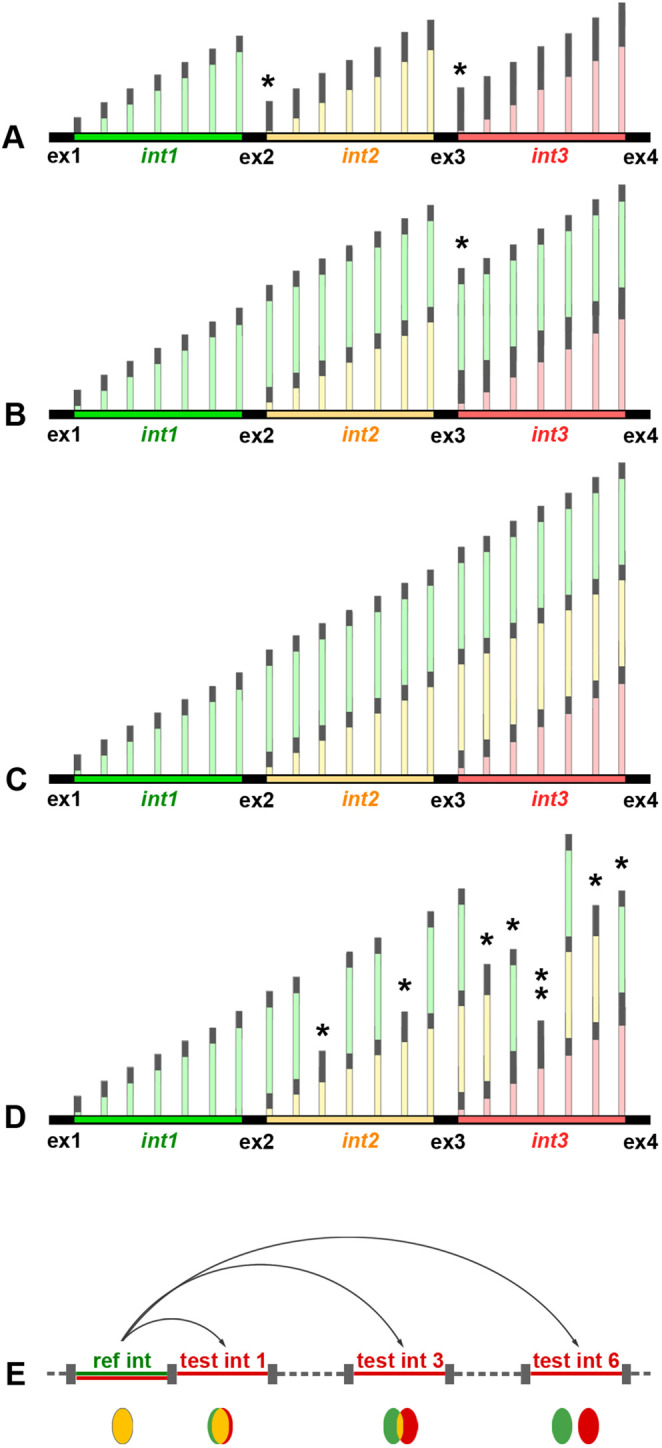
**Schematics of possible splicing scenarios and RNA-FISH experiments for estimation of splicing delay.** (A–D) Schematics of possible splicing scenarios. Strictly, co-transcriptional splicing (A) implies that introns are excised shortly after polymerases cross the 5′ exon border. Singular (B) or consecutive (C) introns are excised synchronously on a certain distance from the intron transcription site. (D) Introns are excised in random nascent transcripts in a non-synchronous manner. Note that B, C and D scenarios are not mutually exclusive. (E) The delay in splicing can be estimated by the degree of colocalization of RNA-FISH signals through comparison of a reference-intron (green) with test-introns (red). High signal colocalization (yellow spot) means delay in intron excision; absence of signal colocalization (separated green and red spots) indicates that the reference intron is already excised from nRNAs decorating the test intron. ex, exons; int, introns; asterisks mark spliced transcripts. Schematics of nRNAs (vertical lines) are not to scale to the gene regions (horizontal lines).

The disproportionality of RNA intron signals suggests that a probe for an intron labels not only nRNAs transcribed from the targeted intron but also nRNAs from downstream regions of the gene. We hypothesized that this extra-labeling of the *Tg* TL could be explained by a delay in splicing along the gene body (Fig. 3A–D). Additionally, the observation that introns of different lengths exhibit similar overlap volumes might indicate distinct splicing dynamics for different introns.

### Unspliced *Tg* introns are detectable by PCR

To confirm instances of splicing delay, we extracted total RNA from mouse thyroids and performed PCR using primers designed for regions spanning intron–exon–intron junctions, located ∼250 bp upstream of the 5′ boundary and 750 bp downstream of its 3′ boundary of an exon. For all four tested regions, the intron–exon–intron sequences were successfully amplified, confirming that splicing of these introns is delayed by at least 0.75 kb (Fig. S2). This finding aligns with previous studies showing that, in some human genes, fully transcribed introns might persist within nascent RNAs for up to 2 kb downstream of the splice junction (Drexler et al., 2020; Sousa-Luis et al., 2021). However, the extent of *Tg* intron signal disproportionality observed by microscopy suggested that the splicing delay during *Tg* transcription is much greater than what is technically detectable via PCR. To address this, we sought to detect *Tg* nascent RNAs at a high-throughput level to examine longer fragments of *Tg* nascent transcripts.

### Nanopore RNA sequencing demonstrates massive retardation of *Tg* introns in nRNAs

Detecting delayed splicing from bulk RNA-seq data is challenging, primarily due to the short length of RNA-seq reads, which are typically insufficient for characterizing single or multiple introns within individual transcripts. Recently, several highly efficient parallel methods for nascent RNA analysis have emerged, including GRO-seq (Core et al., 2008), NET-seq (Mayer and Churchman, 2016), TT-seq (Schwalb et al., 2016) and POINT technology (Sousa-Luis et al., 2021). These techniques require either metabolic labeling or the isolation of chromatin-associated RNA, which presents significant challenges for tissue-derived cells, particularly when the tissue is difficult to dissociate or when cultivating primary cells does not preserve their transcriptional program – both of which apply to the thyroid gland (Ullrich et al., 2023).

Although Nanopore sequencing allows long-read RNA sequencing of nascent transcripts (Garalde et al., 2018) and has been used as a powerful tool for dissecting the intricacies of RNA processing dynamics (Parker et al., 2020; Sousa-Luis et al., 2021), in the case of thyroid tissue, we are unable to draw reliable conclusions, which we tentatively attribute to the small amount of RNA we obtained. According to the manufacturer, to obtain accurate and reliable results with this method, the input RNA should be in the range of 1000 ng of total RNA. Given that thyrocytes comprise only 60% of the thyroid gland and that a single mouse thyroid yields less than 200 ng of total RNA, with only a small proportion representing nascent RNAs, the required quantity of thyroids and mice killed for a single experiment would be unreasonably high. Therefore, we sought an alternative approach to estimate the magnitude of the splicing delay.

**Fig. 4.**
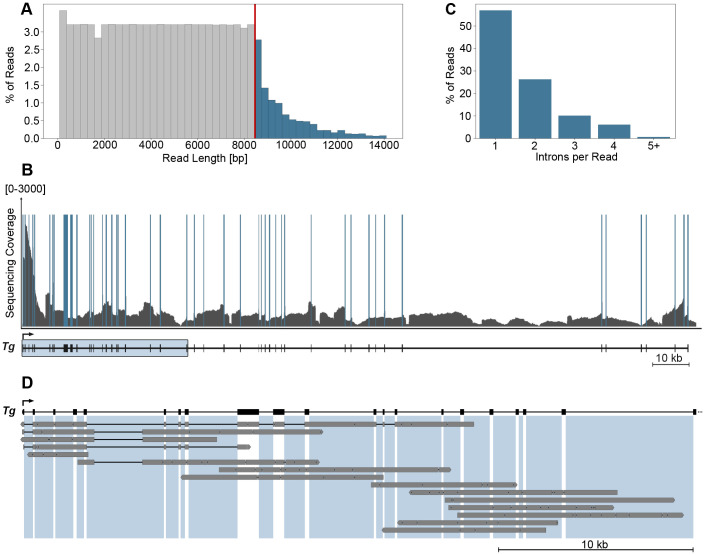
**Nanopore sequencing confirms massive splicing delay of *Tg* introns.** (A) Length distribution of nascent *Tg* RNA sequencing reads containing introns. The *x*-axis represents the length of the individual sequencing reads. Each bar in the histogram corresponds to the frequency of reads with length intervals of 300 bp. The *y*-axis represents the number of reads. The red line designates the length of a fully spliced *Tg* mRNA (8462 bp). (B) Sequencing coverage of nascent RNAs along the *Tg* gene. Solid blue bars depict exons, grey lines depict introns. The *Tg* gene schematic is shown below the graph. The *y*-axis is cut-off at 3000 reads to highlight the intron coverage. (C) Distribution of intron calls per intron containing read. The *x*-axis represents the number of introns contained within individual sequencing reads. Each bar on the histogram represents the frequency of reads. In total, 10,421 intron containing reads aligning to the *Tg* gene were assessed. (D) Graph exemplifying several individual read alignments mapped to the 5′ region of the *Tg* gene, outlined by the box in B. The thick grey lines represent exons and unspliced introns; the thin black lines represent spliced out regions. Areas shaded with light-blue highlight intronic regions within the gene sequence. Sequencing data represents the results of RNA extracted from three mouse thyroids.

To elucidate the dynamics of delayed splicing events, we used an unconventional Nanopore sequencing workflow, enriching the sequencing library for nascent RNAs by depleting polyA RNA from the total RNA isolated from the tissue. Only intron-containing reads were included in the analysis; the length distribution of intron-containing *Tg* RNA reads indicated even sequencing coverage of nascent *Tg* RNAs (Fig. 4A, gray columns). Multiple reads contained one or more fully transcribed introns, whereas others included only an intron region, making it difficult to distinguish between introns that had already been transcribed and those still in the process of transcription. The read lengths spanned up to 8.5 kb, which is consistent with the size of fully spliced and mature *Tg* mRNA. However, a fraction of reads exceeded 8.5 kb, reaching up to 15 kb (Fig. 4A, blue columns), clearly indicating the presence of unspliced introns. Coverage across all intronic regions within the *Tg* gene was comprehensive and uniform (Fig. 4B), highlighting the robustness of our approach in capturing nascent transcripts.

Unsurprisingly, the distribution analysis of intron-containing reads revealed a predominant presence of reads with only a single intron. However, a noticeable fraction of reads incorporated multiple introns, with some containing up to eight introns (Fig. 4C). Recent studies using techniques such as PRO-seq (Reimer et al., 2021) and POINT-seq (Sousa-Luis et al., 2021) have demonstrated that uncleaved transcripts can accumulate at the 3′ end of genes due to the presence of unspliced introns, thus contributing to the population of transcripts without poly(A) [polyA(−)]. Our analysis showed an even distribution of reads containing unspliced introns around the 3′ end of the *Tg* gene (Fig. 4B). Although we detected unspliced transcripts downstream of the *Tg* polyA site, they represented only a small fraction of the polyA(−) transcripts – ∼350 reads out of ∼110,000 total reads aligning to the last *Tg* exon – and therefore did not significantly contribute to the analyzed intron-containing fraction of nascent RNAs.

Examination of individual reads revealed instances where delayed splicing was clearly manifested, with multiple unspliced introns present in a single read, sometimes combined with already spliced introns (Fig. 4D). Our previous Nanopore sequencing data of the poly(A)-enriched mRNA fraction extracted from mouse thyroid tissue showed no intron retention in the polyA(+) *Tg* mRNA fraction (Ullrich et al., 2023). Therefore, we ruled out the possibility that the introns in our reads are due to regulatory intron retention for controlled mRNA export and conclude that our data reflect a splicing delay. Together, our findings demonstrated a delay in splicing across both single and multiple introns within individual *Tg* transcripts; however, due to technical limitations, we were unable to determine the exact duration of the splicing delay for individual introns.

### Pairwise comparison of intron signals confirms splicing delay

We reasoned that the considerable length of *Tg* TLs (Fig. 1A) makes it possible to examine splicing at the single-cell level using RNA-FISH and microscopy. First, given that RNA-FISH does not require denaturation of DNA in tissue sections, probes targeting introns will not hybridize to the gene DNA, ensuring reliable detection of nRNAs. Second, visualizing differentially labeled introns in a pairwise manner would enable a signal comparison between a reference intron and downstream introns (Fig. 3E). We hypothesized that if there is a delay in splicing of an intron, its signal would colocalize with the signal from the subsequent intron or even with subsequent multiple introns. Based on our observation of co-transcriptional splicing on the *Tg* gene at a global scale (Fig. 1D), we anticipated that intron signals would separate as the genomic distance between them increases (Fig. 3E).

**Fig. 5.**
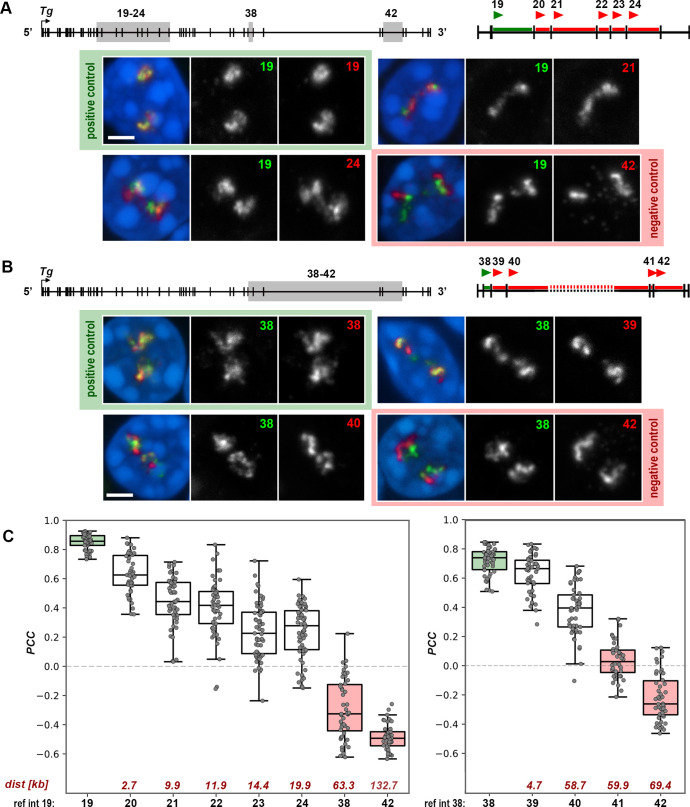
**Pairwise comparison of *Tg* intron signals suggests splicing delay for tens of kilobases.** (A,B) Pairwise comparison of RNA signals between single introns. Regions of intron sampling are marked on gene schematics by grey rectangles. On the magnified views of the sampling regions, green lines and red lines mark the reference intron and test introns, respectively; arrowheads mark 5′ end of introns, for which oligoprobes were designed. Exemplifying images of thyrocytes after RNA-FISH with pairwise intron probes are shown below the schematics. The reference introns 19 (A) and 38 (B) are green; the test introns are red. For clarity, the overlaid RGB images of RNA signals are supplemented with greyscale images. Numbers of introns are indicated on the grey scale panels. Images are projections of short confocal stacks. Scale bars: 2 µm. For more examples of nuclei in this experiment, see Fig. S3. (C) Boxplots showing the PCC values for pairwise intron comparison for regions on A (left) and B (right). Introns are arranged along the *x*-axis according to their genomic positions. Digits below the *x*-axis are intron numbers; digits above the axis are distances to the reference intron (kb). Thick lines within boxes indicate the medians; boxes show 25th and 75th percentiles; whiskers represent the smallest and the largest values within 1.5 times the interquartile range from the quartile 1 and quartile 3 boundaries; gray dots are individual data points. Analysis of 50 nuclei from thyroids of three different mice. Positive and negative controls are highlighted with light-green and light-red colors, respectively.

For this analysis, we used the same regions in the beginning and the end of the gene as in the previous RNA-FISH experiment (Fig. 2; Fig. S1). Within each region, we selected a reference intron at the 5′ end – specifically, introns 19 and 38 – and compared their signals with those of differentially labeled downstream test introns. Additionally, we included positive and negative controls for both regions. For the positive controls, which demonstrate maximally positive correlation, we used the same reference introns labeled with two distinct fluorophores. For the negative controls, we compared the reference intron with downstream introns positioned at a distance of no less than 50 kb (Fig. 5; Fig. S3).

For each comparison, we estimated the colocalization of introns using the Pearson's correlation coefficient (PCC). Reference and test introns were differentially labeled and detected in two spectrally separated channels, thus enabling pixel intensity-based colocalization analysis. This analysis involves evaluating the intensity of each pixel in one channel against the intensity of the corresponding pixel in the other channel, generating a correlation coefficient (Cordelieres and Bolte, 2014; Cordelières and Zhang, 2020). For this purpose, we modified our analysis pipeline to calculate the PCC between signals in two channels within a 3D volume (see Materials and Methods for details). Importantly, the collected image stacks were corrected for both axial and lateral chromatic shifts between the spectrally separated channels. To accomplish this, stacks of 0.5 µm beads labeled with four fluorophores (TetraSpeck™ microspheres) were collected during each acquisition session and processed alongside the samples.

As expected, the signals of both fluorophores in positive controls visually overlap (green fields in Fig. 5A,B) and the mean PCCs for them were close to 1 (Fig. 5C). In contrast, the signals for the negative controls were visually separated (pink fields in Fig. 5A,B), and their PCC values were negative (Fig. 5C). Of note, the anti-correlation does not carry any specific biological meaning but instead indicates signal separation. The mean PCC values for seven pairwise comparisons between the reference and tested introns ranged between those for the positive and negative controls, consistently decreasing as the genomic distances between them increased (Fig. 5C), which well corresponded to the visual estimates (Fig. 5A,B; Fig. S3A,B).

Our microscopy analysis tentatively suggests that intron splicing might be delayed over tens of kilobases, i.e. assuming an average polymerase speed of ∼3.8 kb/min (Singh and Padgett, 2009), for up to tens of minutes after transcription. Undeniably, however, our microscopy approach has its limitations. RNA-FISH detects hundreds of nascent transcripts densely arranged along the *Tg* TLs, making it impossible to identify a single nascent transcript within this mass. Consequently, we cannot determine whether splicing is delayed in all nascent RNAs containing a particular intron. However, based on the bright intron signals, this delay appears to be present in the majority of them.

Furthermore, our analysis involves structures smaller than 0.5–1 µm, which are near the resolution limit of light microscopy. As a result, we cannot completely rule out the possibility that the colocalization of intron signals is influenced, to some degree, by the coiling of the gene axis, which could bring neighboring introns into close proximity and mix their signals. However, our observations of the disproportionality of single intron signals compared to the nearly full-length TL (Fig. 2) suggested that gene axis coiling alone cannot fully explain the phenomenon we observed in the pairwise intron comparison.

### The moderately expressed *Cald1* gene does not display splicing delay

The *Tg* gene is characterized by an exceptionally high expression level of ∼23,000 transcripts per million (TPM) (Leidescher et al., 2022). We hypothesize that this high transcription level might lead to a localized depletion of the splicing machinery, preventing timely spliceosome assembly and thereby causing splicing delays. Unfortunately, we are unable to test this hypothesis directly by manipulating *Tg* expression in a mouse because transcription inhibition results in TLs withdrawal (Leidescher et al., 2022), which would prevent microscopy analysis. Additionally, we cannot analyze other genes with similar length and high expression levels, as we are unaware of any other mammalian genes that meet these criteria.

We reasoned that, as an indirect test, we could examine a gene with a length comparable to that of *Tg* but with lower expression levels to assess whether it also exhibits splicing delay. For this, we selected the *Cald1* gene, which is 177 kb long and expressed in cultured myoblasts at ∼1800 TPM. The expression level of *Cald1* is only ∼8% of that of *Tg* and can be considered moderate. In line with this lower transcription level, the gene forms small, although microscopically resolvable, transcription loops (Leidescher et al., 2022).

**Fig. 6.**
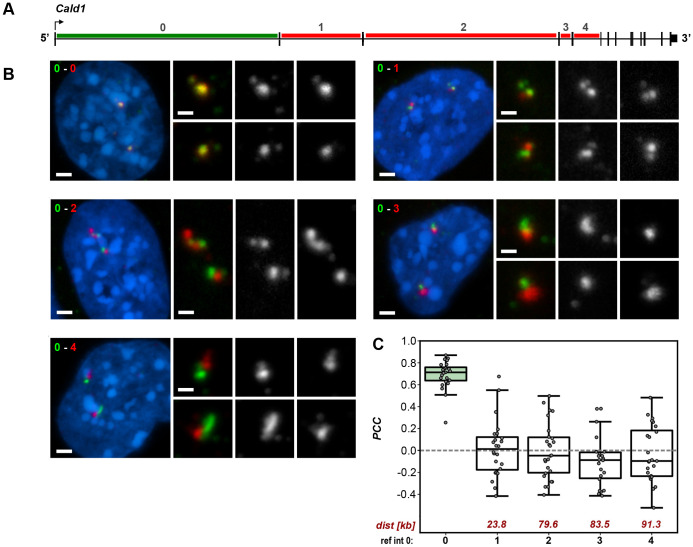
**Splicing delay is not detected in the moderately expressed *Cald1* gene.** (A) Schematics of the *Cald1* gene with marked reference intron (green line) and test introns (red lines). Intron numbers are indicated above the gene axis. (B) Exemplifying images of myoblasts after RNA-FISH with pairwise intron probes. The reference intron 0 is green, the test introns 1–4 are red. Numbers of compared introns are indicated in the upper left corners. Magnified views of the signals are shown on the right and supplemented by grayscale images. Images are projections of short confocal stacks. Scale bars: 2 µm (panels with nuclei); 1 µm (magnified views). (C) Boxplots showing the PCC values for pairwise intron comparison with introns arranged along the *x*-axis according to their genomic positions. Digits below the axis are intron numbers; digits above the axis are genomic distances to the reference intron (kb). Thick lines within boxes indicate the medians; boxes show 25th and 75th percentiles; whiskers represent the smallest and the largest values within 1.5 times the interquartile range from the quartile 1 and quartile 3 boundaries; gray dots are individual data points. Analysis of 25 nuclei from three different batches of cultured cells.

Similar to the *Tg* gene, we tested the splicing dynamics of *Cald1* through pairwise colocalization comparison of intron signals (Fig. 6). The first *Cald1* intron was used as a reference, and colocalization of its signal was measured pairwise with the four subsequent test introns (Fig. 6A). Visual inspection of confocal stacks through the positive control, that is the reference intron labeled with two fluorophores, showed colocalization of signals. The signals for the subsequent test introns, despite small size of the Cald1 TLs, were evidently distinguishable from the reference signal (Fig. 6B). In line with these observations, the PCC values between the reference and test introns were at zero or below (Fig. 6C). Taken together, our data indirectly support the hypothesis that transcription level may influence local splicing dynamics.

### Short and long introns display different splicing dynamics

**Fig. 7.**
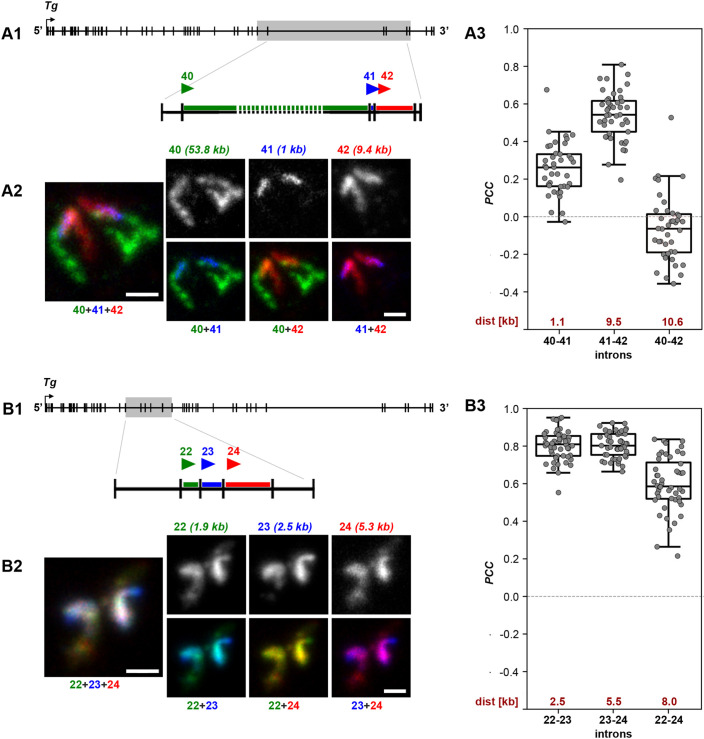
**Long introns exhibit only small delay in splicing.** (A,B) Estimation of colocalization between three consecutive introns that include one long intron (53.8 kb) followed by two short ones (1 and 9.4 kb) (A) and between three consecutive short introns (1.9, 2.5 and 5.3 kb) (B). A1 and B1 are schematics of the gene with highlighted regions of the tested introns also showing the color code. A2 and B2 represent telling examples of intron colocalization. In addition to RGB pairwise overlays and an overlay of all three channels, grayscale images of single introns are shown. Images are projections of several optical sections. Scale bars: 1 µm. Boxplots in A3 and B3 show the PCC values for pairwise intron comparison. Digits below the *x*-axis show intron numbers; digits above X-axis indicate distances between them (kb). Thick lines within boxes indicate the medians; boxes show 25th and 75th percentiles; whiskers represent the smallest and the largest values within 1.5 times the interquartile range from the quartile 1 and quartile 3 boundaries; gray dots are individual data points. Analysis of 50 nuclei from thyroids of three different mice. Note, that by both visual inspection of the RNA-FISH signals and PCC measurement, the longest *Tg* intron 40 shows little correlation with the following small intron 41 and no correlation with intron 42, whereas both short introns 41 and 42 colocalize strongly (A), similar to the three short introns in a different gene region (B).

The proportional size of the *Tg* intron 40 signal compared to the BAC signal (Fig. 2B,C) suggested that there is no significant delay in the splicing of this particular intron. To investigate this further, we labeled three sequential introns – 40 (53.8 kb), 41 (1 kb) and 42 (9.4 kb) – with three distinct fluorophores (Fig. 7A1). This setup allowed for the three pairwise colocalization comparisons of the introns (Fig. 7A2). Consistent with the previous experiments indicating splicing delay in *Tg* introns (Fig. 5), the two sequential introns, 41 and 42, showed a high degree of colocalization. Interestingly, the colocalization between introns 40 and 41 was significantly lower and even became negative between introns 40 and 42 (Fig. 7A3). For comparison, we used three shorter sequential introns, 22 (1.9 kb), 23 (2.5 kb), and 24 (5.3 kb) (Fig. 7B1). As anticipated, colocalization of the three differentially labeled introns resulted in high PCCs for all pairwise comparisons (Fig. 7B2, B3).

The negative correlation between introns 40 and 42, which are separated by only 10.4 kb, contrasts with the data on the colocalization of introns 19–21 and 19–22 – these introns are also separated by 10–12 kb but exhibit a PCC of ∼0.5 (Fig. 5C). The prompt splicing of the longest *Tg* intron 40 suggests that intron length could indeed influence splicing speed. This observation aligns with a recent finding using POINT-nano technology, which demonstrated a positive correlation between intron size and splicing efficiency (Sousa-Luis et al., 2021).

We propose several hypothetical scenarios to explain this phenomenon. First, it is possible that this intron undergoes recursive splicing, requiring less time for complete excision. Although this scenario is plausible, it likely applies only to a minority of transcripts, as we observe the entire intron labeling with the 5′ probe and steadily increasing gradient of nRNAs toward its 3′ end (Fig. 2B). The second scenario considers the pronounced extension of the intron 40 from the main *Tg* transcriptional loop (Fig. 2B; Fig. S1B; Leidescher et al., 2022), positioning the intron in an environment with less competition for splicing machinery. Finally, assuming that the RNAPII transcription speed is ∼4 kb/min (Singh and Padgett, 2009), transcribing the entire 53.8 kb intron would take over 14 min, providing more time to assemble the necessary splicing machinery compared to shorter introns, which are transcribed in seconds to several minutes.

## DISCUSSION

Our microscopy analysis suggests that during transcription of the highly and perpetually upregulated *Tg* gene, splicing of small introns (<10 kb) is delayed over tens of kilobases, a finding confirmed by standard cell population analyses, including PCR and Nanopore sequencing. Intriguingly, the long *Tg* intron (>50 kb) stands out as an exception to this phenomenon, showing no measurable splicing delay. We acknowledge that obtaining a precise estimation of the splicing delay for specific introns within individual transcripts by microscopy is not feasible. First, RNA-FISH generates massive signals from hundreds of nascent RNA transcripts densely decorating *Tg* subregions, rendering individual transcripts indistinguishable. Second, the limited resolution of light microscopy, even with super-resolution techniques (Leidescher et al., 2022), prevents detailed resolution of intron folding and its structure within the *Tg* gene. We tentatively attribute the splicing delay to the high transcriptional activity of the *Tg* gene, which might cause a localized depletion of the splicing machinery across multiple nascent RNA transcripts. As indirect supporting evidence for this hypothesis, we demonstrated that the introns of the *Cald1* gene, a gene of comparable length but with more than 90% lower expression, are excised without a noticeable delay.

Splicing delay might follow different scenarios: (1) only certain introns experience delayed splicing (Fig. 3B); (2) intron sequences persist across several exons before undergoing extensive splicing at a specific time point (Fig. 3C); (3) in a subset of nascent RNAs, introns undergo asynchronous splicing downstream of their transcription sites (Fig. 3D). It is likely that a combination of these scenarios coexists during intense transcription. However, as noted above, the resolution of both Nanopore sequencing and microscopy analysis does not allow us to differentiate between these potential scenarios. For example, we could distinguish between cases illustrated by in Fig. 3A from Fig. 3B or Fig. 3C,D, but not between Fig. 3C and D, because RNA-FISH would yield similar signals in these two case.

Delayed splicing has been demonstrated multiple times (Merens et al., 2024) and, in particular, described for polyadenylated pre-mRNA (Brody et al., 2011; Coté et al., 2024; Drexler et al., 2020; Gaidatzis et al., 2015; Zeng et al., 2022). Analysis of polyA(+) *Tg* RNA fraction, however, has not revealed intron retention (Ullrich et al., 2023) and thus the observed delay in splicing cannot be explained by this phenomenon. Irrespective of the possible scenarios discussed above, delayed splicing provides an explanation for why *Tg* transcription loops extend so profoundly into the nuclear interior. The accumulation of introns in nascent RNAs gradually increases the size of nRNPs over tens of kilobases. Consequently, the increased bulkiness of these nRNPs enhances the stiffness of the gene axis, forcing the gene to extend further into the nuclear space – a phenomenon previously demonstrated in biological experiments involving splicing inhibition and computer polymer simulations (Leidescher et al., 2022).

Altogether, our findings illuminate the intricate and dynamic nature of RNA processing, highlighting underlying complexities in the regulation of splicing events during transcript maturation. Despite technical challenges and certain limitations, we demonstrated that working with tissue samples can be highly rewarding, as it often reveals *in vivo* phenomena and processes that may not be present in cultured cells *in vitro*. Additionally, our work underscores the power of microscopy and image analysis in transcription studies.

## MATERIALS AND METHODS

### Tissue collection and cryosections

Thyroid tissue sampling was executed in accordance with the European Union (EU) directive 2010/63/EU on the protection of animals used for scientific purposes and in compliance with regulations by the respective local Animal Welfare Committees (LMU; Committee on Animal Health and Care of the local governmental body of the state of Upper Bavaria; Germany). Adult male and female CD-1 and C57BL/6 mice were housed in individual cages with free access to food and water on a 12-h-light–12-h-dark cycle at the Biocenter, Ludwig-Maximilians-University of Munich (LMU). Mice were killed by cervical dislocation after IsoFlo (Isofluran, Abbott) narcosis. Freshly dissected tissues were washed with PBS and then fixed with 4% paraformaldehyde (Carl Roth) solution in PBS for 12–20 h. After fixation, thyroids were washed with PBS, cryoprotected in a series of sucrose, and embedded in Tissue-Tek O.C.T. compound freezing medium (Sakura). Blocks were stored at −80°C before cutting into 16–20 µm sections using a cryostat (Leica CM3050S). Cryosections were collected on Superfrost Plus slides (Thermo Fisher Scientific) and stored at −80°C before use.

### Cell culture

The mouse myoblast cell line Pmi28 (kindly donated by Prof. Dr. Cristina Cardoso, Technical University Darmstadt, Darmstadt, Germany) was grown in Nutrient Mixture F-10 Ham (Sigma-Aldrich) supplemented with 20% fetal bovine serum (FBS; Biochrom) and 1% penicillin-streptomycin (Sigma-Aldrich) at 37°C and 5% CO_2_. Cells were subcloned on coverslips pretreated with poly-lysine. After a brief wash with pre-warmed PBS, cells were fixed with 4% paraformaldehyde (Carl Roth) solution in PBS for 10 min, washed with PBS (10 min ×3), permeabilized with 0.5% Triton X-100 in PBS for 10 min, washed with PBS supplemented with 0.01% Tween 20 (10 min×3), equilibrated in 2× SSC (made in-house) and stored in 50% formamide in 2× SSC buffer. The cells were regularly tested for contamination.

### Probes

BAC clones encompassing the *Tg* gene (RP24-229C15, RP23-193A18, RP23-266I10) were selected using the UCSC genome browser and purchased from BACPAC Resources (Oakland children's hospital) as agar stabs (https://bacpacresources.org/). BACs were purified via standard alkaline lysis or the NucleoBond Xtra Midi Kit (Macherey-Nagel), followed by amplification with the GenomiPhi Kit (GE Healthcare) according to the manufacturer's instructions. Amplified BAC DNA was labeled with fluorophores using conjugated fluorophore-dUTPs by nick translation (Cremer et al., 2008). Labeled BAC DNA was ethanol precipitated with 10-fold excess of Cot-1 (1 mg/ml; Invitrogen, 18440-016) and 50-fold excess of salmon sperm DNA (5 µg/µl; Sigma); a pellet was formed by centrifugation at 15,000 ***g*** for 30 min and subsequently dried in a SpeedVak, and dissolved in hybridization mixture containing 50% formamide, 1× SSC and 10% of dextran sulfate.

Genomic coordinates of the studied introns are listed in Table S1. Oligoprobes for introns were generated using SABER-FISH protocol as described in detail previously (Leidescher et al., 2022). Briefly, oligonucleotides targeting intron sequences were designed using Paintshop (Hershberg et al., 2021) and ordered as oligonucleotide pools from Integrated DNA Technologies. The oligonucleotides were remapped if necessary for multi-color imaging and extended to ∼500 nt using a primer exchange reaction (Kishi et al., 2019). Finally, the probes were purified using PCR clean-up columns (Macherey-Nagel).

### FISH

RNA-FISH using BACs was performed on cryosections and cultured myoblasts as previously described (for details, see Eberhart et al., 2012; Solovei and Cremer, 2010). Denatured probes were loaded on sections under small glass chambers or on coverslips with myoblasts and sealed with rubber cement. Denaturation of sections and cells was omitted. Hybridizations were carried out in a water bath at 37°C for 1–2 days. After hybridization, rubber cement and chambers were removed, slides with sections or coverslips with myoblasts were washed with 2× SSC at 37°C, 3×30 min, and then with 0.1× SSC at 60°C 1×7 min. Hybridized SABER probes were detected by incubation with 1 μM fluorescently labeled detection oligonucleotides in PBS for 1 h at 37°C followed by washing with PBS for 10 min.

### Microscopy

Confocal image stacks were acquired using a TCS SP5 confocal microscope (Leica) using a Plan Apo 63×/1.4 NA oil immersion objective and the Leica Application Suite Advanced Fluorescence (LAS AF) Software (Leica). *z* step size was adjusted to an axial chromatic shift and typically was either 200 nm or 300 nm; *xy* pixel size varied from 20 to 60 nm. Chromatic shift was measured using Tetraspeck beads (0.5 µm).

### Image analysis

All image sets (i.e. *z*-stack imaged in two or three spectrally separated channels) for an overlap or correlation experiment were processed automatically with custom Python scripts (v3.8.0; Python Software Foundation), making use of several packages: numpy (Harris et al., 2020), pandas (https://zenodo.org/records/13819579), skimage (Van der Walt et al., 2014), scipy (Virtanen et al., 2020), matplotlib (Hunter, 2007), seaborn (Waskom, 2021), tifffile (https://zenodo.org/records/6795861) and napari (https://napari.org/). The scripts are available upon request. The main steps are summarized below.

#### Calculation of overlapping of FISH signals in 3D

First, each image set was corrected for chromatic shift between the channels. Next, the DAPI signal was binarized using Otsu's method (Otsu, 1975) and cleaned from small artefacts. Subsequently, a watershedding procedure was employed to separate the nucleus in the center of the image from neighboring nuclei. Objects touching the image borders were removed and the object with the biggest volume was retained as the segmented nucleus.

Within the segmented nucleus, the signal of each FISH channel was segmented. First, the volume around each FISH signal was reduced by calculating and thresholding (Otsu) a local entropy image. The resulting mask was applied to the original FISH image, which was then also thresholded using Otsu's method. The number of remaining objects was evaluated and a maximum of two objects (biggest volumes) per FISH channel was retained.

Finally, the overlap between objects in both FISH channels was calculated and compared to the total volume of objects in both FISH channels. Specifically, the signals of single introns were overlapped with a BAC probe delineating most of the gene. To ensure only results from correctly segmented signals remained, image sets where less than 80% of the segmented intron overlapped with the segmented BAC were excluded from the analysis.

#### Calculation of correlation between FISH signals in 3D

For the pixel-based colocalization analysis of FISH signals, images were processed as described above until the FISH segmentation. The volume around the FISH signals was further confined by summing both FISH channels and thresholding the result (Otsu's method) while applying the nuclear mask. This reduced the influence of background fluorescence on colocalization calculations. The resulting mask was cleaned-up from artefacts and objects overlapping with the nuclear border were removed. The FISH signals were smoothed (Gaussian filter, σ=1) before the mask was applied to reduce the value differences between neighboring pixels. Finally, the pixel-wise Pearson correlation coefficient (PCC) between the resulting volumes of both FISH signals was computed. The resulting correlation values were saved in .csv format and plotted using R Statistical Software (https://www.r-project.org/).

### PCR analysis

Dissected thyroids were immediately placed in Lysis Buffer RA1 and homogenized using an ultra-turrax dispersing tool. Total RNA was isolated using the NucleoSpin RNA Kit (Macherey-Nagel). 1 μg of total RNA was reverse transcribed using Maxima H Minus Reverse Transcriptase (Thermo Scientific) with gene-specific primers. PCR was performed in technical and biological triplicates using Phusion Plus polymerase (Thermo Scientific) and subsequently analyzed on a 1% agarose gel. All steps were performed according to the manufacturer's recommendation. Distances between introns were calculated from the 3′ end of a reference intron to the 3′ end of a test intron. All used primers can be found in Table S2.

### Nanopore sequencing

Freshly dissected thyroids were immediately placed in ice-cold TriZol and homogenized using an ultra-turrax dispersing tool. The total RNA pool was depleted of poly(A)+ transcripts using magnetic oligoT-beads (Lexogen). The sequencing library from the remaining RNA was generated using the PCR-cDNA Sequencing Kit (PCB111.24, Oxford Nanopore) and sequenced on a PromethION P24 in a R9.4.1 flowcell. The sequencing data were basecalled using Guppy v6.4.6 and mapped to the mouse genome (mm10) using minimap2 (Li, 2018).

## Supplementary Material

10.1242/joces.263872_sup1 Supplementary information
