# Supplementary material for: Co-transcriptional splicing is delayed in the highly expressed thyroglobulin gene
Source: J Cell Sci. 2025 Mar 19;138(6):jcs263872. doi: 10.1242/jcs.263872 (PMC11959613; doi:10.1242/jcs.263872)
Supplement: Supplementary information [file joces-138-263872-s1.pdf]

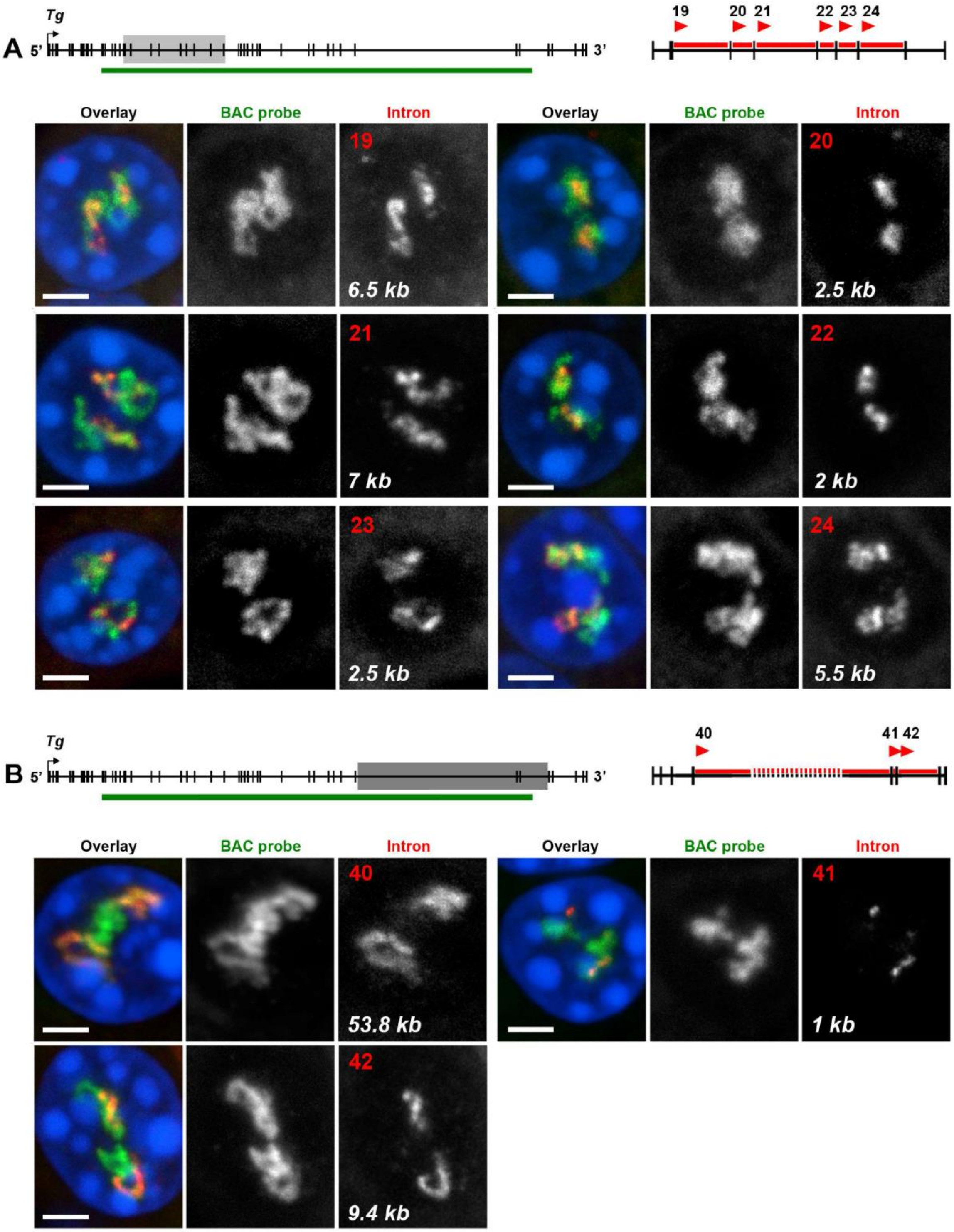

Journal of Cell Science • Supplementary information

Fig. S1. Overlapping between RNA signals of the Tg introns and BAC probe encompassing the middle of the gene. Pairwise comparison of RNA signals of a single intron versus BAC probe (153 kb) in the 5' (A) and 3' (B) gene regions The regions are marked on gene schematics with *light-grey* and *dark-grey* rectangles,respectively.The *green* lines below gene schematics indicate lo- calization of the BAC probe;the *red* arrowheads mark 5'-end of introns with in the indicated regions,for which oligoprobes were designed. Images are projections of confocal stacks through thyrocytes after RNA-FISH with pairwise probes (BAC, *green;* intrans,*red).* For clarity, RGB images are appended with grey scale images of RNA signals. Numbers and lengths of intrans are indicated in the grey scale intron panels. Scale bars: *2 µm.*

Journal of Cell Science • Supplementary information

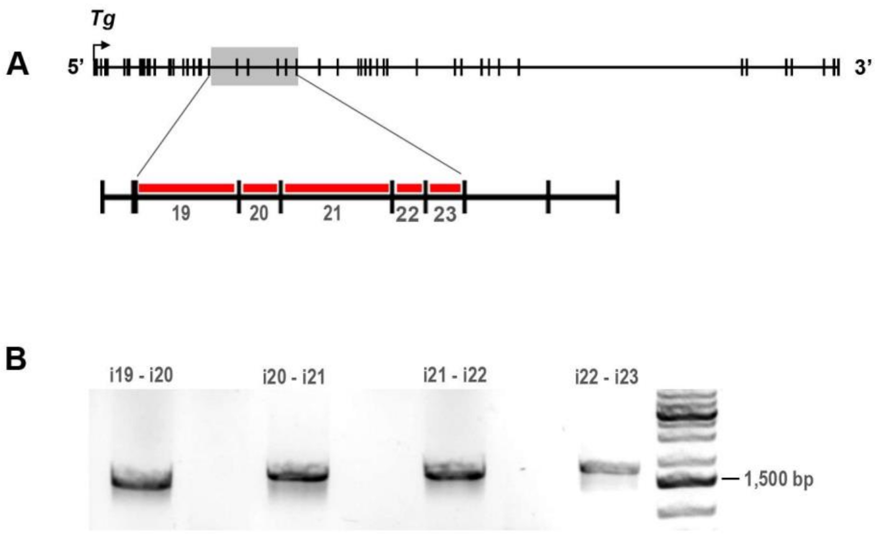

Fig. S2. Unspliced Tg introns are detectable by PCR

A, Gene schematics showing localization of the tested *intron-exon-intron* sequences. In the enlarged view,the used intrans are marked with red lines. **B,** PCR with primers designed from both sides of the tested exons: ca. 250 bp upstream of exon 5' border and ca. 750 bp downstream of its 3' border. For all four tested exons, the *intron-exon-intron* sequences were successfully amplified.

Journal of Cell Science • Supplementary information

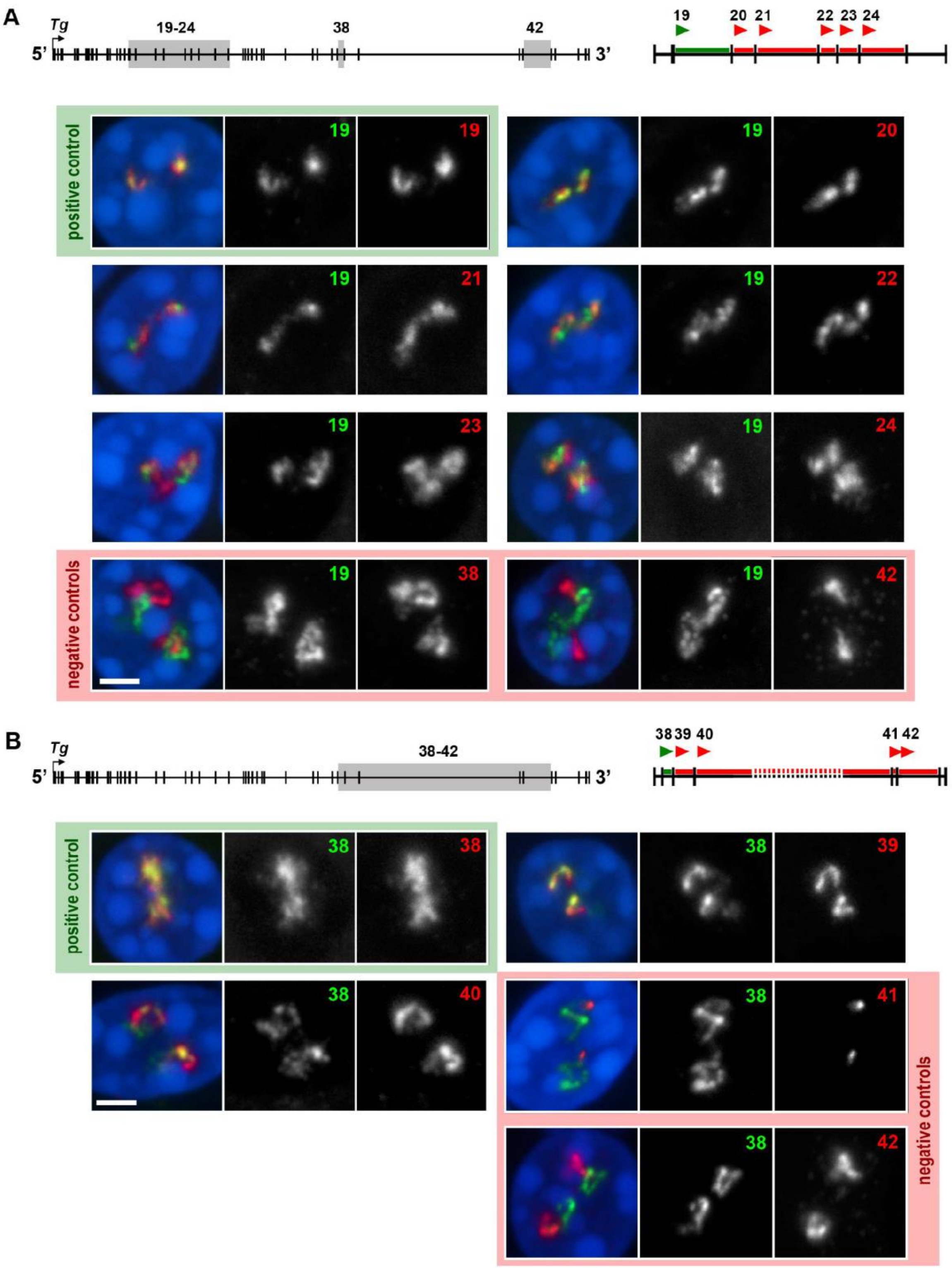

Journal of Cell Science • Supplementary information

Fig. S3. Pairwise comparison of Tg intron signals.

A,B, exemplifying images of thyrocytes after RNA-FISH with pairwise comparison of RNA signals between single intrans. Two regions with detected intrans at the 5' end (A) and 3' end (B) are marked on gene schematics by *grey* rectangles.On the close-ups of the sampling regions, *green lines* and *red lines* mark the reference-intrans and testintrans,respectively; arrowheads mark 5'-end of intrans, for which oligoprobes were designed. Positive and negative controls are highlighted by *green* and *red* fields, respectively . For clarity,RGB images of overlaid RNA signals are appended with grey scale images Numbers of intrans are indicated on the grey scale panels Images are proiections of short confocal stacks. Scale bars: *2 µm.*

Journal of Cell Science • Supplementary information

Table S1. Genomic coordinates of the investigated introns

gene	chrom	intron number	start	end	length [bp]
Thyroglobulin	chr15	Intron 19	66698655	66705204	6549
Thyroglobulin	chr15	Intron 20	66705354	66707872	2518
Thyroglobulin	chr15	Intron 21	66708043	66715070	7027
Thyroglobulin	chr15	Intron 22	66715187	66717127	1940
Thyroglobulin	chr15	Intron 23	66717243	66719649	2406
Thyroglobulin	chr15	Intron 24	66719758	66725089	5331
Thyroglobulin	chr15	Intron 38	66766212	66768535	2323
Thyroglobulin	chr15	Intron 39	66768695	66773278	4583
Thyroglobulin	chr15	Intron 40	66773481	66827262	53781
Thyroglobulin	chr15	Intron 41	66827427	66828398	971
Thyroglobulin	chr15	Intron 42	66828566	66837941	9375
Caldesmon1	chr6	Intron 0	34598665	34662127	63462
Caldesmon1	chr6	Intron 1	34662216	34685962	23746
Caldesmon1	chr6	Intron 2	34686074	34741788	55714
Caldesmon1	chr6	Intron 3	34741935	34745618	3683
Caldesmon1	chr6	Intron 4	34746015	34753442	7427

Table S2. Used RT & PCR primers

Use	Sequence Name	Sequence
PCR	Tg i19 Fwd	TTC TCA GCT AGC TCC TTA ACT TT
PCR	Tg i20 short Rev	CAT CCA GCT GCT GTA AGA TAG T
PCR	Tg i20 long Rev	GAC AGA CTG ACA CTC ACA GAA G
PCR	Tg i20 Fwd	GGG TCT CAG TGG TGT TCT TT
PCR	Tg i21 short Rev	GGA GAT CTT TCC ATT TCA CTG TTC
PCR	Tg i21 long Rev	ACA CCT GTG TAC TGA GGA AAT G
PCR	Tg i21 Fwd	GTT ACA CTG CCG AGT CTG AA
PCR	Tg i22 short Rev	CTC CTA ACA CTC AGT CCA ACA C
PCR	Tg i22 long Rev	TCA TGG TCC CTC CTC CTA AA
PCR	Tg i22 Fwd	GTG TGT GTG TGT GTG TGA ATA G
PCR	Tg i23 short Rev	ACT CTT CGG TTG GTG GAT TAG
PCR	Tg i23 long Rev	GAT TGG GCT GGA ACC ATA AGA
PCR	Tg i23 Fwd	TCC TCA GTT CTC CTT CTC CAT A
PCR	Tg i24 short Rev	TCC TTG GTT CCT GCT GTA TG
PCR	Tg i24 long Rev	CAT CCA GAT CCA CTC CCT TTC
Reverse Transcription	RT Tg 1	GCA AGA TCA GCC ATT AG
Reverse Transcription	RT Tg 2	TAA AGT CGT GGG AAA TAA G
Reverse Transcription	RT Tg 3	CTC CTG AAC ACT TCT TAT AC
Reverse Transcription	RT Tg 4	TTC ATA GCA CTG GAC TGT CTT C
Reverse Transcription	RT Tg 5	GTG AGT GGT AGG CAC TGA TAT T

Journal of Cell Science • Supplementary information
